# Pharmacokinetics and Tissue Distribution of Itampolin A following Intragastric and Intravenous Administration in Rats Using Ultra-High-Performance Liquid Chromatography–Tandem Mass Spectrometry

**DOI:** 10.3390/molecules29112652

**Published:** 2024-06-04

**Authors:** Qi Sun, Jingwei Liang, Qingyu Zhang, Xuezhen Wang, Nan Zhao, Fanhao Meng

**Affiliations:** 1 School of Pharmacy, China Medical University, Shenyang 110122, China; qsun@cmu.edu.cn (Q.S.); zhangqingy999@163.com (Q.Z.); w306213644@163.com (X.W.); 2 School of Pharmacy, Hainan Medical University, Haikou 570100, China; jwliang@hainmc.edu.cn

**Keywords:** itampolin A, UHPLC-MS/MS, pharmacokinetics, tissue distribution

## Abstract

Itampolin A, a natural brominated tyrosine alkaloid isolated from the sponge *Iotrochota purpurea*, has been shown to have good inhibitory effects in lung cancer cells as a p38α inhibitor. A simple, sensitive, and reliable ultra-high-performance liquid chromatography–tandem mass spectrometry (UHPLC-MS/MS) method has been established, validated, and applied to the study of the pharmacokinetics and tissue distribution of itampolin A following intragastric and intravenous administration. Itampolin A and theophylline (internal standard, IS) were extracted by the simple protein precipitation technique using methanol as the precipitating solvent. Chromatographic separation was achieved by using the optimized mobile phase of a 0.1% formic acid aqueous solution and acetonitrile in the gradient elution mode. Itampolin A and IS were detected and quantified using positive electrospray ionization in the multiple reaction monitoring mode with transitions of *m*/*z* 863.9 → 569.1 for itampolin A and *m*/*z* 181.1 → 124.1 for IS, respectively. The assay exhibited a linear dynamic range of 1–1600 ng/mL for itampolin A in biological samples and the low limit of quantification was 1 ng/mL. Non-compartmental pharmacokinetic parameters indicated that itampolin A was well-absorbed into the systemic circulation and rapidly eliminated after administration. The apparent distribution volume of itampolin A was much higher after intragastric administration than that after intravenous administration. A tissue distribution study showed that itampolin A could be detected in different tissues and maintained a high concentration in the lung, which provided a material basis for its effective application in lung cancer. The pharmacokinetic process and tissue distribution characteristics of imtapolin A were expounded in this study, which can provide beneficial information for the further research and clinical application of itampolin A.

## 1. Introduction

As the earliest multicellular organisms in the world, sponges are the most plentiful source of biological active substances in marine invertebrates [1,2,3]. Majority of the natural products isolated from sponges belong to brominated tyrosine derivatives, which exhibit extensive biological activities including antiviral [4,5], antibacterial [6], anti-inflammatory [7], cytotoxic [8], antifungal [9], and anticancer activities [10,11]. Itampolin A is a natural brominated tyrosine alkaloid, which was first isolated from the sponge *Iotrochota purpurea* [12]. In the previous work, our team first achieved the total synthesis of itampolin A, and reported its significant anticancer activity against non-small-cell lung cancer [13]. Additionally, our study demonstrated that the anticancer mechanism of itampolin A was associated with its binding to the DFG-out conformation of p38α, and then decreasing the phospho-p38α expression [14].

It is generally known that the drug quantification in vivo is crucial for the evaluation of a novel therapeutic agent [15]. Considering that itampolin A is a novel potential anticancer drug, it is essential to investigate its pharmacokinetics and tissue distribution properties, which will be beneficial for exploring its mechanism of action and facilitating the further research of the novel natural p38α inhibitor. However, to our best acquaintance, there has been no analytical method reported for the quantification of itampolin A in any biological matrix. Thus, in order to reveal the pharmacokinetics and tissue distribution properties of itampolin A in vivo, it is highly necessary and meaningful to establish a sensitive and efficient method for the determination of itampolin A in plasma and various tissues.

Nowadays, UHPLC-MS/MS has become one of the most efficient methods for analyzing complex biological samples due to its high selectivity and sensitivity [16,17,18]. Therefore, a simple, sensitive, and reliable UHPLC-MS/MS method was developed and validated to measure itampolin A in rat plasma and tissues for the first time in this paper. This method was then successfully applied to investigate the pharmacokinetics and tissue distribution of itampolin A in rats after intragastric and intravenous administration, in order to elucidate the plasma concentration and evaluate the tissue distribution characteristics. The results of our assay will provide a foundation support for the further research and clinical application of itampolin A.

## 2. Results and Discussion

### 2.1. Method Optimization

#### 2.1.1. Optimization of Extraction Method

Protein precipitation and liquid–liquid extraction were considerably compared to select a suitable clean-up method for biological samples. For convenience, efficiency, and accuracy, the protein precipitation method was applied for sample preparation in the present study. This method could meet the analytical requirement for the detection of the extraction recovery and matrix effect. Acetonitrile and methanol were compared to choose a suitable solvent for protein precipitation, and the results indicated that deproteinization with methanol was the best choice, yielding a higher extraction recovery of more than 88% for itampolin A.

#### 2.1.2. Optimization of UHPLC-MS/MS Conditions

The objectives of method optimization were to achieve a sensitive quantification for itampolin A in a reasonable elution time. To obtain a better performance in peak shape, separation, and signal response, the chromatographic conditions including column type, mobile phase composition, and flow rate were optimized. Various analytical columns were tried, and, ultimately, an Agilent ZORBAX Eclipse Plus C18 (100 mm × 3.0 mm, 1.8 µm) column was applied due to the reproducible response and good peak symmetry. The selection of the mobile phase is important to improve the peak shape and increase the signal response. Gradient elution was tested with methanol-water, acetonitrile-water, and acetonitrile-water containing 0.1% formic acid. Using acetonitrile as the organic phase resulted in a decreased running time, and the peak area of itampolin A was almost 2.5 times higher with acetonitrile-water as the mobile phase than that with methanol-water. Moreover, whether using methanol-water or acetonitrile-water, there was a slight tailing in the alkaloid peak and such an effect was difficult to be eliminated by altering the liquid chromatogram condition. The addition of formic acid in the mobile phase could provide a weak electrolyte effect and delivered a good peak shape of itampolin A in the positive ion mode [19]. Finally, the acetonitrile-0.1% formic acid water was selected as the mobile phase with a flow rate of 0.3 mL/min. Under these conditions, itampolin A and theophylline (internal standard, IS) were eluted at 5.03 and 3.62 min, respectively, with a total run time of 6.5 min.

The optimization of MS/MS detection was started by choosing the electrospray ionization (ESI) mode. The ESI source with positive or negative ionization were tested for the determination. The mass spectra showed that the more stable and higher response could be observed in the positive ionization mode. In order to obtain a better signal intensity and wide compound overage for quantification, the MS/MS ion transitions were then monitored in the multiple reaction monitoring (MRM) mode [20]. The protonated molecular ions [M + H]^+^ of itampolin A and IS were *m*/*z* 863.9 and *m*/*z* 181.1 in the Q1 full-scan mass spectrum. Given a certain amount of collision energy, the most abundant and constant ions in the product ion scan were *m*/*z* 569.1 and *m*/*z* 124.1 for itampolin A and IS, respectively. Hence, the MRM transitions for itampolin A and IS were determined as *m*/*z* 863.9 → 569.1 and *m*/*z* 181.1 → 124.1, respectively, which were employed for the quantitation. The parameters were also optimized to acquire a higher sensitivity and response, which were shown in Section 3.3.

### 2.2. Method Validation

#### 2.2.1. Specificity

**Figure 1:**
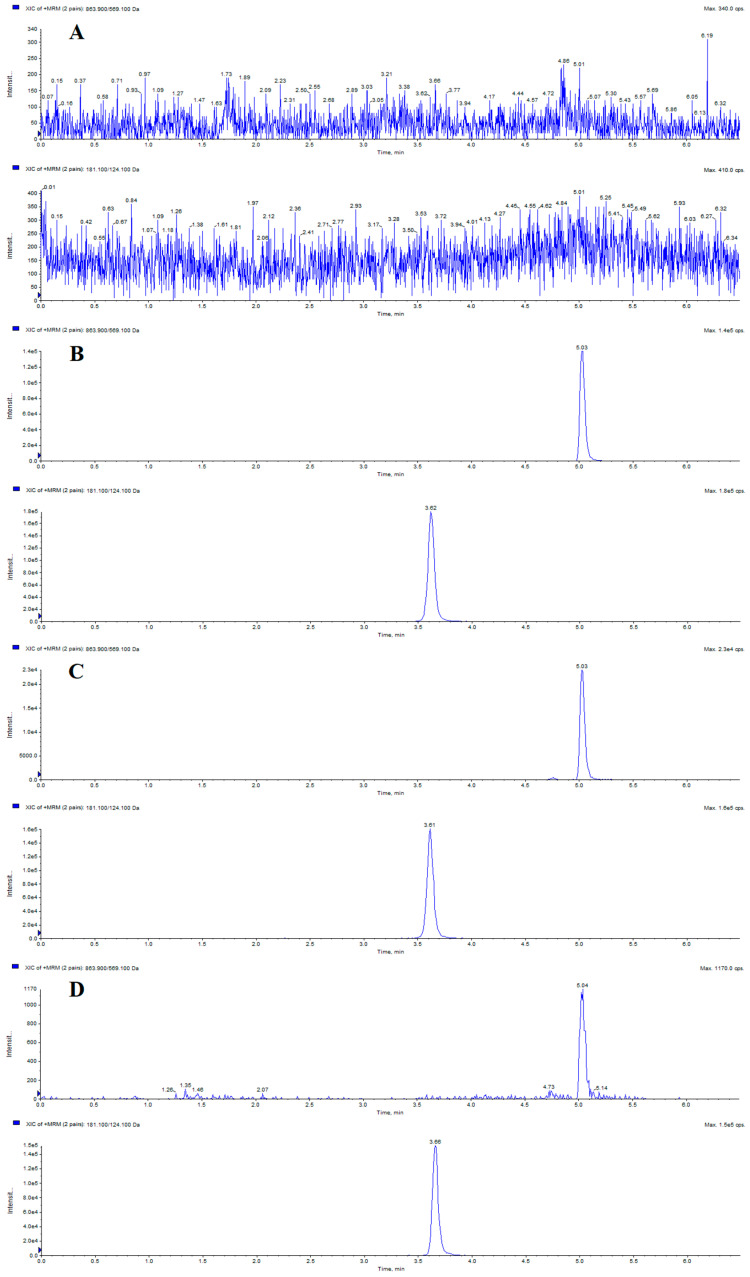
Representative chromatograms of itampolin A and theophylline (IS): (**A**) blank plasma sample; (**B**) blank plasma sample spiked with itampolin A (5.03 min) and IS (3.62 min); (**C**) real plasma sample after i.g. administration of itampolin A; and (**D**) plasma sample spiked with itampolin A at LLOQ.

Representative MRM chromatograms involving a blank plasma sample, blank plasma sample spiked with itampolin A and IS, real plasma sample after intragastric administration of itampolin A, and plasma sample spiked with itampolin A at the lower limit of quantification (LLOQ) were shown in Figure 1. The retention times of itampolin A and IS were 5.03 and 3.62 min, respectively. No interfering peaks were observed at itampolin A and IS retention times or within a 10% retention window thereof under these chromatographic conditions, which indicated that there was no apparent endogenous interference for the determination of itampolin A and IS in biological samples.

#### 2.2.2. Calibration Curve and LLOQ

The calibration curves were constructed by plotting the peak area ratios of each analyte to IS (y) versus the corresponding concentration (x) using the weighted (1/x^2^) least square linear regression model. As shown in Table 1, the linear concentration range was 1–1600 ng/mL for both plasma and tissue samples, with the coefficient values all being more than 0.993. The LLOQ of itampolin A was 1.0 ng/mL.

#### 2.2.3. Precision and Accuracy

The accuracy and precision of itampolin A assessed by analyzing the LLOQ and quality control (QC) samples at different concentrations were presented in Table 2. The precision values expressed as the relative standard deviation (RSD) were all within 12.4% and the accuracy values expressed as the relative error (RE) ranged from −11.9% to 8.2%, indicating that the method was accurate and reproducible for the measurement of itampolin A in rat plasma and tissues.

#### 2.2.4. Extraction Recovery and Matrix Effect

The extraction recovery and matrix effect of itampolin A measured at four levels were shown in Table 3. The extraction recoveries of itampolin A in rat plasma and tissues were no less than 87.8%, suggesting that the selected method of extraction was highly efficient. Meanwhile, the matrix effect values ranged from 85.2% to 113.9%, suggesting that the endogenous matrix had no significant impact on the measurement of itampolin A in biological samples.

#### 2.2.5. Stability

The stability of itampolin A under different storage conditions was investigated at three QC levels and the results were summarized in Table 4. Itampolin A was stable during its processing procedures (placed at room temperature for 4 h and kept in the autosampler for 24 h) since the deviation in concentration was within ±15% from nominal values. Moreover, no effect on the quantification of itampolin A was observed during three freeze–thaw cycles and long-term storage (placed at −80 °C for 30 days).

### 2.3. Dose Selection

Different doses were selected in different routes of administration. The dose selection for intragastric administration was based on our previous pharmacodynamic studies and the concentrations of 10 mg/kg, 25 mg/kg, and 50 mg/kg were finally selected. Since the solubility of itampolin A in normal saline is small, a relatively low dose of 5 mg/kg for intravenous administration was selected.

### 2.4. Pharmacokinetic Study

**Figure 2:**
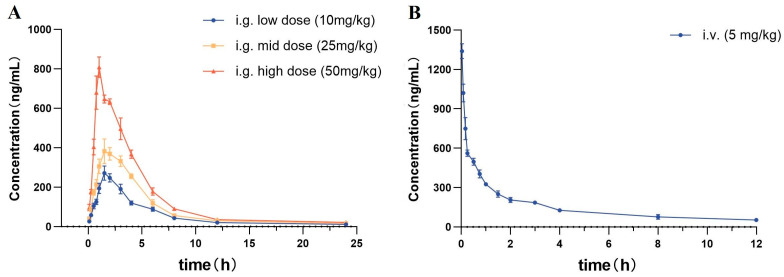
The plasma-concentration–time curve in rats by (**A**) given i.g. dose of 10, 25, and 50 mg/kg of itampolin A (*n* = 6) and (**B**) given i.v. dose of 5 mg/kg of itampolin A (*n* = 6).

The validated method for the quantification of itampolin A in rat plasma was applied to the pharmacokinetic study in rats after the intragastric administration of 10, 25, and 50 mg/kg itampolin A; and the intravenous administration of 5 mg/kg itampolin A. Based on our present knowledge, this was the first study reporting the pharmacokinetic characteristics of itampolin A in vivo. The mean plasma-concentration–time curves of itampolin A were shown in Figure 2, while the main pharmacokinetic parameters were listed in Table 5.

#### 2.4.1. Intragastric Administration PKs

As shown in Table 5, upon the intragastric administration of 10, 25, and 50 mg/kg itampolin A, the T_max_ were less than 1.7 ± 0.2 h, and the t_1/2_ were from 2.1 ± 0.2 h to 2.6 ± 0.3 h. It was suggested that the oral administration of itampolin A could be rapidly absorbed into plasma and quickly eliminated, which might be related to the large polarity of itampolin A containing multiple halogen and hydroxyl groups [21]. Meanwhile, the AUC_0–t_, AUC_0–∞_ and C_max_ versus the itampolin A dose profile were linear with correlation coefficients of 0.999, 0.998, and 0.981, respectively, showing dose-dependent PKs. According to the formula F = Dose_iv_ × AUC_ig_/(Dose_ig_ × AUC_iv_) × 100%, the absolute bioavailability of itampolin A were calculated to be 18.8%, 13.7%, and 11.2% for oral doses of 10, 25, and 50 mg/kg, respectively, indicating the relatively low oral absorption of the drug in rats. The low bioavailability of itampolin A might be related to the high molecular weight and poor water solubility of the compound. In addition, the Vd values were from 42.2 ± 2.9 to 44.6 ± 2.8 L/kg, indicating that itampolin A exhibited a more obvious tissue uptake after oral administration, which was confirmed by the subsequent tissue distribution study. The CL were from 11.1 ± 0.5 to 12.9 ± 0.8 L/h/kg, which indicated that the CL of itampolin A did not increase with the dose.

#### 2.4.2. Intravenous Administration PKs

As shown in Table 5, upon the intravenous administration of 5 mg/kg itampolin A, the T_max_ was 0.03 h and the C_max_ was 1339 ± 52.2 ng/mL, indicating that itampolin A could be quickly detected in the plasma. The t_1/2_ was 2.6 ± 0.2 h, which is comparable to that of intragastric administration, implying that the drug had a relatively stable and rapid elimination process in rats. Itampolin A had an apparent Vd of 6.6 ± 0.4 L/kg which suggested that there was no obvious accumulation in tissues after intravenous administration. The pharmacokinetic results have demonstrated the MRT and CL of 4.8 ± 0.3 h and 1.8 ± 0.1 L/h/kg, respectively. Moreover, the AUC_0–t_ and AUC_0–∞_ were found to be 978.0 ± 26.2 ng·h/mL and 2829.9 ± 109.7 ng·h/mL, respectively.

### 2.5. Tissue Distribution Study

**Figure 3:**
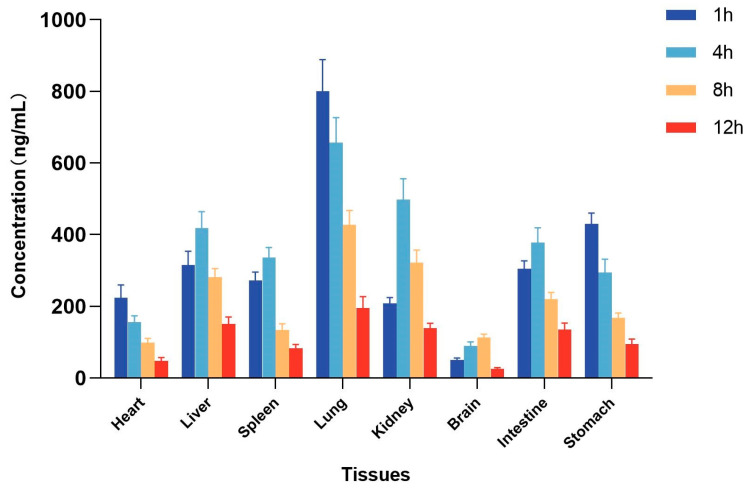
Tissue distribution in rats after intragastric administration of 25 mg/kg itampolin A (mean ± SD, *n* = 5).

The method for the quantification of itampolin A in rat tissues was applied to the tissue distribution study in rats after the intragastric administration of 25 mg/kg itampolin A. The concentrations of itampolin A in various tissues were calculated by standard curves, and the mean-tissue-concentration–time columnar chart was shown in Figure 3. The concentrations in the heart, liver, spleen, lung, kidney, brain, intestine, and stomach were measured within 12 h, respectively. Different levels of itampolin A were detected in various tissues, indicating that it could be widely distributed in rat tissues after oral administration, consistent with the results of the PKs study. The highest concentration of itampolin A was observed in the lung (800.6 ± 79.0 ng/mL), followed by the kidney (497.7 ± 51.9 ng/mL), stomach (429.6 ± 27.2 ng/mL), liver (418.8 ± 40.3 ng/mL), and intestine (378.2 ± 36.8 ng/mL). The concentration in tissues decreased obviously after 8 h, suggesting that no long-term accumulation occurred to itampolin A. In terms of distribution characteristics, the concentration of itampolin A in lung tissue was the highest and the elimination was the slowest, indicating an obvious accumulation in the lung. We speculate that the highest distribution of itampolin A in the lung laid the material foundation for its effective application in lung cancer [13]. Meanwhile, the higher concentration in the kidney and liver suggested the possibility of metabolism through the liver and excretion through the kidney, which provided a reference for our further study on the excretion pathway and metabolites of itampolin A [22]. In addition, itampolin A had a relatively high concentration in the stomach and intestine, which was probably related to the administration route. In contrast, itampolin A was less likely to cause drug accumulation and immune side effects in the heart and spleen due to the low distribution level and rapid elimination. At the same time, the lower concentration in the brain indicated that itampolin A might not easily pass through the blood–brain barrier and produced pharmacological effects.

According to the results of tissue distribution, itampolin A was widely distributed in the tissues and exhibited high concentrations in the lung after oral administration. The excessive tissue distribution led to low plasma exposure. The calculated F value for oral administration was low, but this does not mean that itampolin A is not suitable for oral administration. We will focus on the bioavailavility improvement of itampolin A in future studies.

## 3. Materials and Methods

### 3.1. Reagents and Materials

Itampolin A (>98% purity) was synthesized by Professor Jingwei Liang, School of Pharmacy, Hainan Medical University and its structure was identified by detailed NMR and MS analysis [12,13]. Theophylline (>98% purity) as the internal standard was purchased from Sigma-Aldrich (Shanghai, China). HPLC-grade acetonitrile, methanol, and formic acid were supplied by Fisher Scientific (Tustin, CA, USA). Ultra-pure water was prepared by a Milli-Q purification system (Bedford, MA, USA). Other reagents used were of analytical grade and purchased through regular channels.

### 3.2. Animals

Sprague–Dawley rats (250 ± 20 g) were supplied from the Experimental Animal Centre of China Medical University (Shenyang, China) and housed under unified conditions. All of the animal experiments were conducted in compliance with the Guidelines for Care and Use of Laboratory Animals of China Medical University and the protocols were approved by the Animal Ethics Committee of the institution (code number: CMU2023205). The rats were raised in a standard environment with a temperature of 24 ± 2 °C, relative humidity of 55 ± 10%, and 12 h light/12 h dark cycles. These rats were acclimatized to the environment for at least one week before the experiment, provided free access to drinking water, and fed during this period. Twenty-four SD rats, which were divided into four groups (six rats per group), received 5 mg/kg itampolin A by intravenous administration or 10, 25, and 50 mg/kg itampolin A by intragastric administration for the pharmacokinetics experiments. Twenty SD rats (five rats for each time point) received 25 mg/kg itampolin A by intragastric administration for the tissue distribution study. 

### 3.3. Instruments and UHPLC-MS/MS Conditions

An Agilent series 1290 UHPLC system (Agilent Technologies, Palo Alto, CA, USA) coupled to an AB 3500 triple quadrupole mass spectrometer (AB Sciex, Concord, ON, Canada) via an ESI source was used for sample separation and detection. The instrument control and data acquisition were performed on the Analyst software 1.6.3 (AB Sciex, Concord, ON, Canada). 

The chromatographic separation was achieved on an Agilent ZORBAX Eclipse Plus C_18_ column (100 mm × 3.0 mm, 1.8 µm) and eluted with a mobile phase consisting of acetonitrile (A) and 0.1% formic acid aqueous solution (B) as follows: 0–0.2 min, 10–10% A; 0.2–2.3 min, 10–95% A; 2.3–5.4 min, 95–95% A; and 5.5–6.5 min, 10–10% A. The flow rate was 0.3 mL/min, the injection volume was 10 µL, and the column temperature was set at 30 °C.

**Figure 4:**
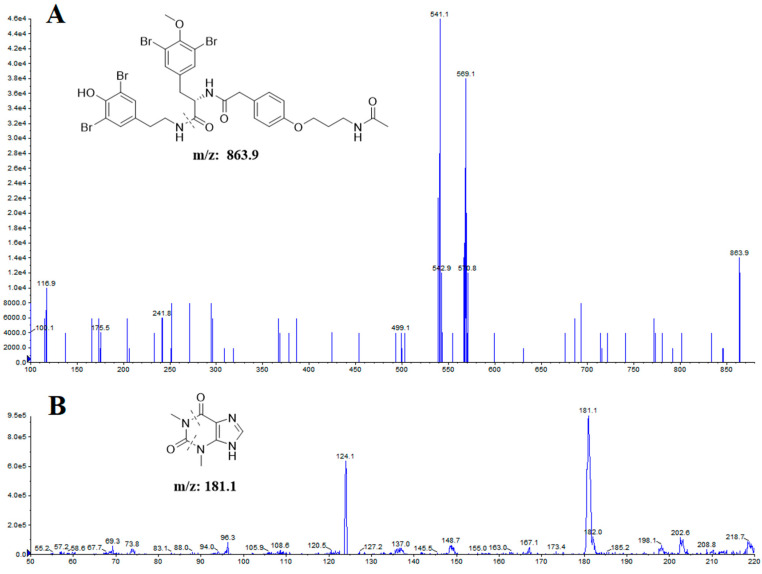
Full-scan product ion spectra of itampolin A (**A**) and IS (**B**).

The mass spectrometric detection was conducted under multiple reaction monitoring in a positive ionization mode using the following conditions: ion spray voltage of 5.5 kV, turbo spray temperature of 500 °C, collision gas at 8 psi, curtain gas at 35 psi, nebulizer gas at 35 psi, and heater gas at 30 psi. Nitrogen was used in all cases. The optimization of the MS transition for quantitation were accomplished as *m*/*z* 863.9 → 569.1 for itampolin A, and *m*/*z* 181.1 → 124.1 for IS (Figure 4). The precursor ion-production ion transition, corresponding declustering potential (DP), and collision energy (CE) were summarized in Table 6.

### 3.4. Preparation of Calibration Standards and Quality Control (QC) Samples

A concentration of 1.0 mg/mL itampolin A stock solution was prepared by dissolving 10.0 mg of itampolin A in 10 mL of methanol. Then, the stock solution was serially diluted to the desired concentrations as working standard solutions. Calibration curves were prepared by spiking the appropriate standard solution into a certain volume of blank biological substrates, as described in “3.5. Sample Preparation” below. Effective concentrations of itampolin A in samples were 1, 4, 8, 16, 32, 80, 320, 640, and 1600 ng/mL for plasma and tissue homogenates (heart, liver, spleen, lung, kidney, brain, intestine, and stomach). A concentration of 1.0 mg/mL theophylline stock solution was prepared by dissolving 10.0 mg of theophylline in 10 mL of methanol. Then, the stock solution was further diluted with methanol to prepare the IS working solution (200 ng/mL). The concentrations of QC samples in plasma, heart, liver, spleen, lung, kidney, brain, intestine, and stomach were 3, 40, and 1280 ng/mL of itampolin A, which were prepared in the same procedure as that used for the preparation of calibration samples. All stock and working solutions were kept at 4 °C away from light and were brought to room temperature before analysis.

### 3.5. Sample Preparation

The simple protein precipitation extraction technique using methanol as the precipitating solvent was applied to extract itampolin A and IS from biological samples [23,24]. The 100 µL of plasma sample, 50 µL of IS working solution, and 200 µL of methanol were added in 500 µL microcentrifuge tubes to precipitate proteins. After vortexing for 1 min, the samples were centrifuged at 12,000 rpm for 10 min to remove the denatured protein. Subsequently, the supernatant was transferred into another tube and evaporated to dryness with a stream of nitrogen at 35 °C. The remains were reconstituted in 100 µL of initial mobile phase, briefly vortexed, and centrifuged at 12,000 rpm for 10 min. Then, 10 µL of the supernatant was transferred to sampling vials for the UHPLC-MS/MS system.

Various tissues (heart, liver, spleen, lung, kidney, brain, intestine, and stomach) were harvested and washed with ice-cold 0.9% sodium chloride solution to remove the superficial blood. After being blotted dry with filter paper, each tissue sample weighed accurately was homogenized in 0.9% sodium chloride solution (1:5, *w*/*v*). Then, 100 µL of tissue homogenate was taken and processed further like the plasma sample. 

### 3.6. Sample Preparation

In accordance with the guidelines of the Food and Drug Administration for bioanalytical method validation [25], the UHPLC-MS/MS method was validated in terms of specificity, linearity, precision, accuracy, extraction recovery, matrix effect, and stability [26,27]. 

#### 3.6.1. Specificity

The specificity was evaluated by comparing the chromatograms of blank biological samples, biological samples spiked with itampolin A and IS, and the real biological samples obtained after the administration of itampolin A, so as to check if the potential endogenous substances interfered with the determination of itampolin A and IS.

#### 3.6.2. Calibration Curve and LLOQ

Calibration curves were created by plotting the peak area ratios of itampolin A to IS (y) against the concentration of that calibration standard (x) by weighted (1/x^2^) least square linear regression. The calibration standards of itampolin A were in the concentration range of 1–1600 ng/mL for both plasma samples and tissue samples. The LLOQ was defined as the lowest concentration of the calibration curve that can be quantitated with both the precision and accuracy within ±20%.

#### 3.6.3. Precision and Accuracy

The intra- and inter-day precision and accuracy were assessed by analyzing the LLOQ sample (1 ng/mL) and QC samples (3, 40, 1280 ng/mL) on one day and three consecutive days, using calibration curves established daily (*n* = 6). Precision was calculated as the RSD of measured concentrations, while accuracy was measured as the RE. The acceptable values of RSD and RE for this validation were within ±15%.

#### 3.6.4. Extraction Recovery and Matrix Effect

For extraction recovery and matrix effect analysis, LLOQ and three QC concentrations (1, 3, 40, and 1280 ng/mL) were applied. Extraction recovery was determined by contrasting the peak areas of itampolin A in the extracted samples with those in the non-processed samples (*n* = 5). The matrix effect was evaluated by comparing the peak ratios of itampolin A dissolved in blank biological matrices versus those dissolved with mobile phase instead of biological matrices (*n* = 5). In common, both the precision of extraction recovery and matrix effect should be no more than 15%.

#### 3.6.5. Stability

The stability of itampolin A was investigated by analyzing five replicates of the samples at three QC levels (3, 40, and 1280 ng/mL) under different storage conditions: (1) 4 h exposure at room temperature, (2) 24 h storage in the autosampler at 4 °C, (3) three freeze–thaw cycles, and (4) storage at −80 °C for 30 days. The samples were considered stable if the average percentage concentration deviation was within 15% of the actual value.

### 3.7. Pharmacokinetic Study

#### 3.7.1. Intragastric Administration

For both intragastric and intravenous administration, itampolin A was dissolved in polyethylene glycol (PEG)-saline (1:3, PEG: 380–420) for the preparation of dosing solutions. Eighteen Sprague–Dawley rats were divided into three groups randomly (six rats each). The rats of each group were weighed, and then given a single dose of 10 mg/kg, 25 mg/kg, and 50 mg/kg itampolin A via intragastric administration, respectively. Blood samples (200 µL) were obtained from orbital vein into heparinized centrifuge tubes at 0.083, 0.25, 0.5, 0.75, 1, 1.5, 2, 3, 4, 6, 8, 12, and 24 h after drug administration.

#### 3.7.2. Intravenous Administration

Six Sprague–Dawley rats were weighed, and then given a single dose of 5 mg/kg of itampolin A via intravenous administration. Blood samples (200 µL) were obtained from orbital vein into heparinized centrifuge tubes at 0.033, 0.083, 0.167, 0.25, 0.5, 0.75, 1, 1.5, 2, 3, 4, 8, and 12 h after drug administration. The blood samples obtained from both intragastric and intravenous administration were immediately separated by centrifugation at 12,000 rpm for 10 min, and then the supernatant plasma was gathered and stored at −20 °C prior to analysis. 

### 3.8. Tissue Distribution Study

Twenty Sprague–Dawley rats were randomly divided into four groups (five rats each) and received a single dose of 25 mg/kg of itampolin A via intragastric administration. The rats were executed by cervical dislocation at 1, 4, 8, and 12 h. Afterwards, various tissue samples, including heart, liver, spleen, lung, kidney, brain, intestine, and stomach, were collected into centrifuged tubes, and then processed with the methods described in “3.5. Sample Preparation”. 

### 3.9. Data Analysis

The pharmacokinetic parameters were calculated by non-compartmental model using the DAS 3.2.8 software [28]. All data were presented as mean ± SD. 

## 4. Conclusions

In this study, we first established an accurate, sensitive, and reliable UHPLC-MS/MS method for the quantification of itampolin A in rat plasma and tissues. With the advantage of the simple sample preparation procedure, short analysis time, and high sensitivity, this method was successfully applied to evaluate the pharmacokinetics and tissue distribution profiles of itampolin A in rats after intragastric and intravenous administration. The pharmacokinetic results suggested that itampolin A was well-absorbed into the systemic circulation and rapidly eliminated after administration. The apparent Vd of itampolin A was much higher after intragastric administration than after intravenous administration, indicating a much wider distribution and accumulation of itampolin A in the tissues after oral administration. Meanwhile, itampolin A could be detected in almost all of the tissues examined, indicating that it could be widely distributed in rat tissues after oral administration. Moreover, the concentration of itampolin A in the lung and kidney was relatively high, suggesting that the lung and kidney might be the targeting organs of itampolin A. These results were beneficial to the further investigation of itampolin A and provided useful information for its clinical application.

## Figures and Tables

**Table 1:** Calibration curves, correlation coefficients, linear ranges, and LLOQs of itampolin A in biological samples.

Samples	Calibration Curves	Correlation Coefficients (r^2^)	Linear Ranges (ng/mL)	LLOQs (ng/mL)
Plasma	Y = 0.0750 + 0.00569x	0.9966	1–1600	1
Heart	Y = 0.0560 + 0.00604x	0.9985	1–1600	1
Liver	Y = 0.0337 + 0.00736x	0.9939	1–1600	1
Spleen	Y = 0.0945 + 0.01077x	0.9945	1–1600	1
Lung	Y = 0.0047 + 0.00877x	0.9986	1–1600	1
Kidney	Y = 0.0530 + 0.00803x	0.9983	1–1600	1
Brain	Y = 0.0271 + 0.01345x	0.9959	1–1600	1
Intestine	Y = 0.0163 + 0.00723x	0.9975	1–1600	1
Stomach	Y = 0.0586 + 0.00905x	0.9969	1–1600	1

**Table 2:** Precision and accuracy of itampolin A in biological samples (*n* = 6).

Samples	Spiked CONC (ng/mL)	Measured CONC (ng/mL)	Accuracy (RE, %)	Intra-Day Precision (RSD, %)	Inter-Day Precision (RSD, %)
Plasma	1	0.93 ± 0.08	−7.3	9.3	6.2
	3	3.25 ± 0.20	8.2	8.0	5.8
	40	41.53 ± 2.65	3.8	4.7	6.6
	1280	1227.34 ± 69.64	−4.1	9.5	4.9
Heart	1	1.02 ± 0.04	2.2	3.7	2.2
	3	3.24 ± 0.34	8.0	11.1	3.1
	40	36.39 ± 4.70	−9.0	4.5	13.6
	1280	1345.66 ± 63.85	5.1	4.4	6.6
Liver	1	0.96 ± 0.05	−4.5	5.3	7.3
	3	3.18 ± 0.37	6.0	12.4	2.9
	40	41.30 ± 3.52	3.3	9.0	2.2
	1280	1329.90 ± 82.87	3.9	5.3	10.8
Spleen	1	0.93 ± 0.10	−7.1	11.3	9.0
	3	3.11 ± 0.29	3.6	9.8	5.0
	40	41.64 ± 3.41	4.1	8.6	4.5
	1280	1310.37 ± 52.98	2.4	3.9	4.9
Lung	1	1.05 ±0.06	4.6	5.6	4.8
	3	2.77 ± 0.32	−7.8	12.0	8.1
	40	42.88 ± 3.77	7.2	8.5	10.6
	1280	1233.68 ± 84.06	3.6	6.7	7.9
Kidney	1	1.03 ± 0.06	3.1	5.6	8.4
	3	3.09 ± 0.28	2.8	9.8	2.6
	40	36.89 ± 3.71	−7.8	10.6	4.9
	1280	1334.12 ± 97.11	4.2	7.1	8.4
Brain	1	1.06 ± 0.08	5.7	7.2	8.1
	3	3.07 ± 0.24	2.3	8.1	6.1
	40	41.72 ± 3.01	4.3	7.5	5.0
	1280	1234.17 ± 69.70	−3.6	5.5	7.0
Intestine	1	1.08 ± 0.07	8.2	6.8	7.0
	3	2.79 ± 0.26	−6.9	9.4	9.1
	40	40.81 ± 2.16	2.0	5.3	5.2
	1280	1320.34 ± 70.49	3.2	5.4	4.7
Stomach	1	1.03 ± 0.05	3.2	4.4	5.2
	3	2.64 ± 0.31	−11.9	12.2	7.6
	40	39.18 ± 3.15	−2.1	8.3	5.7
	1280	1333.97 ± 59.48	4.2	4.7	2.4

**Table 3:** Extraction recovery and matrix effect of itampolin A in biological samples (*n* = 5).

Samples	Spiked CONC (ng/mL)	Matrix Effect		Extraction Recovery	
		Mean ± SD (%)	RSD (%)	Mean ± SD (%)	RSD (%)
Plasma	1	105.2 ± 6.0	5.7	97.3 ± 6.6	6.8
	3	101.8 ± 4.4	4.4	104.0 ± 6.6	6.4
	40	85.2 ± 4.9	5.7	99.0 ± 4.7	4.7
	1280	96.7 ± 3.5	3.6	102.7 ± 3.7	3.6
Heart	1	94.0 ± 4.5	4.8	102.1 ± 4.3	4.2
	3	98.8 ± 3.4	3.4	104.9 ± 2.3	2.2
	40	102.3 ± 2.7	2.6	89.7 ± 4.7	5.3
	1280	101.9 ± 7.0	6.9	95.5 ± 6.3	6.6
Liver	1	92.2 ± 7.8	8.5	101.8 ± 5.6	5.5
	3	100.8 ± 4.2	4.2	103.7 ± 4.3	4.2
	40	97.4 ± 5.8	5.9	98.8 ± 1.8	1.8
	1280	105.3 ± 7.0	6.6	107.5 ± 8.5	7.9
Spleen	1	106.4 ± 5.2	4.9	96.8 ± 4.2	4.3
	3	98.4 ± 6.9	7.0	103.0 ± 9.3	9.0
	40	106.2 ± 2.8	2.7	88.0 ± 4.5	5.1
	1280	99.4 ± 5.2	5.2	94.7 ± 6.0	6.3
Lung	1	97.8 ± 9.5	9.7	104.5 ± 8.7	8.3
	3	94.6 ± 5.9	6.2	96.9 ± 7.6	7.8
	40	113.9 ± 3.6	3.2	101.7 ± 4.7	4.6
	1280	96.1 ± 8.0	8.4	103.2 ± 3.8	3.7
Kidney	1	96.9 ± 6.7	6.9	96.4 ± 4.8	5.0
	3	102.7 ± 6.2	6.0	102.8 ± 6.9	6.7
	40	104.8 ± 2.7	2.6	92.3 ± 5.3	5.8
	1280	99.0 ± 5.1	5.2	104.2 ± 3.1	2.9
Brain	1	100.9 ± 4.7	4.7	92.4 ± 3.2	3.4
	3	97.8 ± 1.3	1.3	98.7 ± 5.6	5.6
	40	112.2 ± 7.6	6.8	92.5 ± 7.2	7.8
	1280	99.4 ± 3.7	3.7	101.8 ± 3.2	3.2
Intestine	1	97.6 ± 6.2	6.3	87.8 ± 5.3	6.0
	3	101.2 ± 3.2	3.2	111.6 ± 2.7	2.4
	40	100.7 ± 7.5	7.4	96.4 ± 4.5	4.6
	1280	94.1 ± 3.6	3.8	99.5 ± 2.1	2.1
Stomach	1	99.5 ± 2.4	2.4	107.1 ± 4.1	3.9
	3	110.6 ± 4.7	4.1	105.9 ± 3.7	3.5
	40	93.3 ± 4.4	3.9	97.9 ± 5.7	5.8
	1280	97.9 ± 5.5	5.6	101.5 ± 8.8	8.7

**Table 4:** Stability of itampolin A in biological samples under different storage conditions (*n* = 5).

Samples	Spiked CONC (ng/mL)	Short-Term (at Room Temperature for 4 h)	Autosampler 4 °C for 24 h	Three Freeze–Thaw Cycles	Long-Term (at −80 °C for 30 Days)
Plasma	3	103.9 ± 8.0	97.3 ± 5.5	106.8 ± 8.8	90.5 ± 6.7
	40	100.1 ± 4.1	96.6 ± 6.3	95.3 ± 8.3	93.6 ± 8.2
	1280	97.8 ± 4.1	98.3 ± 2.9	110.6 ± 5.6	95.8 ± 8.4
Heart	3	97.7 ± 3.6	87.2 ± 7.2	94.3 ± 6.5	103.3 ± 6.8
	40	100.3 ± 2.6	103.9 ± 3.5	108.3 ± 9.7	97.4 ± 5.3
	1280	89.3 ± 5.3	94.0 ± 7.3	98.9 ± 4.9	95.3 ± 5.4
Liver	3	98.2 ± 7.5	96.3 ± 8.2	103.3 ± 4.7	93.7 ± 5.1
	40	93.5 ± 2.5	98.8 ± 4.8	96.4 ± 8.1	87.1 ± 6.8
	1280	102.9 ± 6.5	113.6 ± 4.2	95.1 ± 9.2	97.9 ± 5.2
Spleen	3	96.2 ± 2.2	103.8 ± 8.5	98.4 ± 6.6	89.5 ± 8.9
	40	100.6 ± 3.8	105.8 ± 3.6	112.2 ± 6.5	102.9 ± 8.7
	1280	98.4 ± 2.1	101.2 ± 4.2	92.4 ± 7.8	104.1 ± 5.2
Lung	3	108.7 ± 5.1	102.5 ± 9.3	95.9 ± 7.2	95.6 ± 4.9
	40	103.1 ± 3.3	99.3 ± 6.9	86.0 ± 4.2	106.7 ± 8.8
	1280	101.2 ± 2.9	98.5 ±3.1	91.4 ± 5.9	96.4 ± 4.8
Kidney	3	98.6 ± 4.0	93.0 ± 8.9	97.1 ± 6.7	89.1 ± 7.1
	40	106.8 ± 7.3	100.7 ± 7.6	98.4 ± 5.0	97.3 ± 8.5
	1280	99.8 ± 8.1	92.7 ± 4.2	98.8 ± 3.6	110.4 ± 5.9
Brain	3	112.6 ± 3.9	98.3 ± 5.8	99.5 ± 6.3	94.3 ± 8.7
	40	99.9 ± 10.9	108.0 ± 4.0	93.7 ± 7.8	95.0 ± 2.9
	1280	99.7 ± 2.9	96.8 ± 8.3	104.3 ± 2.6	94.6 ± 5.7
Intestine	3	100.1 ± 6.1	99.5 ± 5.3	108.7 ± 2.9	96.3 ± 7.9
	40	101.3 ± 2.3	106.4 ± 8.9	98.3 ± 5.0	85.9 ± 7.4
	1280	99.7 ± 3.3	102.3 ± 4.1	107.5 ± 8.2	89.0 ± 7.9
Stomach	3	100.6 ± 7.6	87.5 ± 3.3	103.2 ± 4.2	92.6 ± 6.5
	40	99.0 ± 4.3	104.8 ± 7.8	114.8 ± 6.6	95.6 ± 5.8
	1280	99.1 ± 4.2	98.5 ± 2.6	97.7 ± 4.7	94.1 ± 6.1

**Table 5:** The main pharmacokinetic parameters after intragastric administration of 10, 25, and 50 mg/kg itampolin A; and intravenous injection of 5 mg/kg itampolin A (mean ± SD, *n* = 6).

Pharmacokinetic Parameters	i.g. Low Dose (10 mg/kg)	i.g. Mid Dose (25 mg/kg)	i.g. High Dose (50 mg/kg)	i.v. (5 mg/kg)
C_max_ (ng/mL)	283.1 ± 20.0	412.3 ± 13.7	807.1 ± 36.9	1339.0 ± 52.2
T_max_ (h)	1.7 ± 0.2	1.7 ± 0.2	0.9 ± 0.1	0.03 ± 0.0
t_1/2_ (h)	2.6 ± 0.3	2.3 ± 0.2	2.1 ± 0.2	2.6 ± 0.2
k_e_ (h^−1^)	0.27 ± 0.03	0.30 ± 0.02	0.33 ± 0.03	0.27 ± 0.02
AUC_0–t_ (ng·h/mL)	938.9 ± 34.9	1536.1 ± 44.9	2640.1 ± 44.6	978.0 ± 26.2
AUC_0–∞_ (ng·h/mL)	1077.8 ± 55.8	1943.8 ± 116.0	3194.7 ± 94.7	2829.9 ± 109.7
MRT_0–∞_ (h)	4.6 ± 0.5	4.2 ± 0.3	3.7 ± 0.2	4.8 ± 0.3
CL (L/h/kg)	11.1 ± 0.5	12.9 ± 0.8	12.4 ± 0.4	1.8 ± 0.1
Vd (L/kg)	42.2 ± 2.9	43.9 ± 1.7	44.6 ± 2.8	6.6 ± 0.4

**Table 6:** Optimized mass spectrometry conditions for the determination of itampolin A and IS.

Analytes	Transitions (*m*/*z*)	Declustering Potential (V)	Collision Energy (eV)
itampolin A	863.9 → 569.1	148	30.9
IS	181.1 → 124.1	70	26.6

## Data Availability

The data presented in this study are available on request from the corresponding author. The data are not publicly available due to privacy restrictions (containing information that could compromise the privacy of research participants).
